# The *piggyBac*-Based Gene Delivery System Can Confer Successful Production of Cloned Porcine Blastocysts with Multigene Constructs

**DOI:** 10.3390/ijms17091424

**Published:** 2016-08-30

**Authors:** Masahiro Sato, Kosuke Maeda, Miyu Koriyama, Emi Inada, Issei Saitoh, Hiromi Miura, Masato Ohtsuka, Shingo Nakamura, Takayuki Sakurai, Satoshi Watanabe, Kazuchika Miyoshi

**Affiliations:** 1 Section of Gene Expression Regulation, Frontier Science Research Center, Kagoshima University, Kagoshima 890-8544, Japan; 2 Laboratory of Animal Reproduction, Faculty of Agriculture, Kagoshima University, Kagoshima 890-0065, Japan; k7504084@kadai.jp (Ko.M.); k3326891@kadai.jp (M.K.); kmiyoshi@agri.kagoshima-u.ac.jp (Ka.M.); 3 Department of Pediatric Dentistry, Graduate School of Medical and Dental Sciences, Kagoshima University, Kagoshima 890-8544, Japan; inada@dent.kagoshima-u.ac.jp; 4 Division of Pediatric Dentistry, Department of Oral Health Sciences, Course for Oral Life Science, Graduate School of Medical and Dental Sciences, Niigata University, Niigata 951-8514, Japan; isaito@dent.niigata-u.ac.jp; 5 Department of Regenerative Medicine, Basic Medical Science, School of Medicine, Tokai University, Kanagawa 259-1193, Japan; rascal511520531@yahoo.co.jp; 6 Division of Basic Molecular Science and Molecular Medicine, School of Medicine, Tokai University, Kanagawa 259-1193, Japan; masato@is.icc.u-tokai.ac.jp; 7 The Institute of Medical Sciences, Tokai University, Kanagawa 259-1193, Japan; 8 Division of Biomedical Engineering, National Defense Medical College Research Institute, Saitama 359-8513, Japan; snaka@ndmc.ac.jp; 9 Department of Cardiovascular Research, Graduate school of Medicine, Shinshu University, Nagano 390-8621, Japan; tsakurai@shinshu-u.ac.jp; 10 Animal Genome Research Unit, Division of Animal Science, National Institute of Agrobiological Sciences, Ibaraki 305-8602, Japan; kettle@affrc.go.jp

**Keywords:** drug selection, porcine embryonic fibroblasts, multiple transgenes, *piggyBac*, transposon, somatic cell nuclear transfer

## Abstract

The introduction of multigene constructs into single cells is important for improving the performance of domestic animals, as well as understanding basic biological processes. In particular, multigene constructs allow the engineering and integration of multiple genes related to xenotransplantation into the porcine genome. The *piggyBac* (*PB*) transposon system allows multiple genes to be stably integrated into target genomes through a single transfection event. However, to our knowledge, no attempt to introduce multiple genes into a porcine genome has been made using this system. In this study, we simultaneously introduced seven transposons into a single porcine embryonic fibroblast (PEF). PEFs were transfected with seven transposons containing genes for five drug resistance proteins and two (red and green) fluorescent proteins, together with a *PB* transposase expression vector, pTrans (experimental group). The above seven transposons (without pTrans) were transfected concomitantly (control group). Selection of these transfected cells in the presence of multiple selection drugs resulted in the survival of several clones derived from the experimental group, but not from the control. PCR analysis demonstrated that approximately 90% (12/13 tested) of the surviving clones possessed all of the introduced transposons. Splinkerette PCR demonstrated that the transposons were inserted through the TTAA target sites of *PB*. Somatic cell nuclear transfer (SCNT) using a PEF clone with multigene constructs demonstrated successful production of cloned blastocysts expressing both red and green fluorescence. These results indicate the feasibility of this *PB*-mediated method for simultaneous transfer of multigene constructs into the porcine cell genome, which is useful for production of cloned transgenic pigs expressing multiple transgenes.

## 1. Introduction

Methods that allow for the coordinated expression of multiple genes in eukaryotic cells have the potential to significantly enhance our ability to genetically engineer the porcine genome. Such methods could have a diverse range of applications, including genetic improvements to domestic stocks, xenotransplantation, investigations of developmental processes, and the development of animal models for human diseases. In pig-to-human xenotransplantation, introducing several xenotransplantation-related genes into the pig genome to avoid possible immunological attack from the host is a long-standing goal [1].

Pronuclear injection of transgene-containing DNA into fertilized eggs is one of the primary methods used for the production of genetically engineered piglets. However, simultaneous introduction of multigene constructs using this method is problematic, because it is difficult to place multiple expression units into a single construct and, even if successful, the expression of one or more expression units is usually suppressed, most likely owing to gene silencing [2]. Lavitrano and colleagues recently succeeded in introducing multiple transgenes into the pig genome using sperm-mediated gene transfer technology [3]. They observed transgene expression in the resulting piglets. However, this technology is not widely used, probably because of the technical difficulties associated with the technique. In this context of genetically engineered animals, it is preferable to employ the somatic cell nuclear transfer (SCNT) method, despite its very low efficiency [4]. This is because (1) it is possible to introduce multiple gene constructs into cells prior to SCNT; (2) the properties of genetically engineered cells (e.g., transgene expression levels) are easy to analyze; and (3) cells expressing high levels of transgene can be easily isolated [5]. The application of other pre-existing selectable markers would allow us to realize such a concept. In this case, it is desirable to obtain transfectants carrying multigene constructs through a single transfection event, since multiple transfections cause deleterious effects on the normal function of a cell, which in turn causes frequent failures in the SCNT-mediated production of cloned piglets.

The *piggyBac* (*PB*) system derived from the cabbage looper moth *Trichoplusia ni* [6] is an emerging technology for efficient genetic modification of mammalian cells [7,8]. During transposition, the *PB* transposase recognizes transposon-specific inverted terminal repeat sequences (ITRs) located on both ends of the transposon vector and efficiently integrates transgenes into the genome at TTAA nucleotide elements [8,9]. *PB* has been employed for a variety of applications including in vitro transfection in various mammalian cells [10,11,12,13], generation of transgenic mice [10], in vivo gene transfer in mice [14], gene discovery via insertional mutagenesis [15], and production of inducible pluripotent stem (iPS) cells [16,17,18,19]. It is also a useful tool for obtaining stable transfectants from a small number (5.7 × 10^4^) of hard-to-transfect cells [20]. Such applications have opened new areas of research that can lead to the development of new therapeutic strategies for human diseases. However, the *PB*-mediated gene delivery system results in random integration of transgenes, leading to occasional transgene silencing, insertional mutagenesis, and positional variegation, probably as a result of transgene silencing [21,22]. These properties are especially important when this vector system is used for therapeutic gene transfer applications.

The aim of this study was to demonstrate the ability of this *PB*-based gene delivery system to allow simultaneous introduction of multigene constructs into the genome of porcine cells through a single transfection event. As a proof-of-concept, porcine embryonic fibroblasts (PEFs) were co-transfected with seven *PB* transposons (donor vectors) and a *PB* transposase expression vector (helper vector), and then selected in the presence of 5 selection drugs. The emerging drug resistant cells were assessed for expression of fluorescence and the presence of multigene constructs integrated into their genome as well as their ability to develop in vitro into cloned embryos.

## 2. Results

### 2.1. Experiment 1

**Figure 1:**
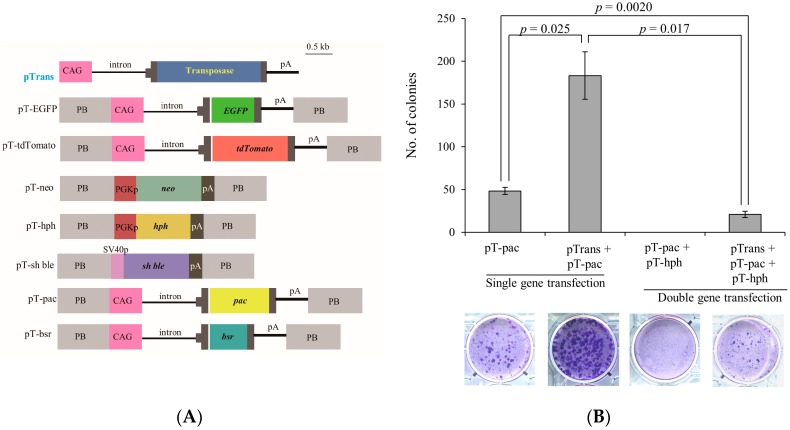
(**A**) Schematic representation of selectable marker expression vectors. Plasmid backbone is not shown in this figure. CAG, cytomegalovirus enhancer + chicken β-actin promoter; pA, poly(A) sites; *hph*, hygromycin phosphotransferase gene; PGKp, mouse phosphoglycerate kinase promoter; *neo*, neomycin resistance gene; *pac*, puromycin-*N*-acetyltransferase gene; *bsr*, blasticidin S deaminase gene; SV40p, SV40 early promoter; *PB*, acceptor site in *piggyBac* system; and *Sh ble*, a protein that binds to zeocin and prevents it from binding DNA; and (**B**) beneficial effects of *piggyBac*-based gene delivery for efficient acquisition of stable transfectants. PEFs were transfected with a single *PB* vector (pT-pac) in the presence or absence of a transposase expression vector, pTrans (pT-pac vs. pTrans + pT-pac in “single gene transfection”), as described in the Materials and Methods. Similarly, they were transfected with double *PB* vectors (pT-pac + pT-hph) in the presence or absence of pTrans (pT-pac + pT-hph vs. pTrans + pT-pac + pT-hph in “double gene transfection”). After drug selection, emerging colonies were counted by staining with Giemsa. Photographs taken after Giemsa staining are shown above each column, together with the number of colonies generated.

We transfected PEFs with single or double *PB* vectors with pTrans, a *PB* transposase expression vector (Figure 1A), to test gene transfer efficiency in the *PB*-based gene delivery system. As controls, *PB* vectors (without pTrans) were concomitantly introduced as described in the Materials and Methods. The gene transfer efficiency was evaluated by calculating the number of emerging stable transfectants after drug selection. The results are shown in Figure 1B. As expected, transfection with a single *PB* vector (pT-pac) + pTrans yielded 176 colonies, but with pT-pac alone resulted in only 45 colonies, indicating approximately four-fold higher gene transfer efficiency in this *PB*-based system. Transfection with double *PB* vectors (pT-pac + pT-hph) + pTrans yielded 22 colonies, whereas the double *PB* vectors alone failed to generate any viable colonies. Thus, gene transfer efficiency in transfectants with double *PB* vectors was reduced approximately seven-fold compared to that observed in transfectants with only a single *PB* vector. Given these results, we concluded that the *PB* system confers higher gene transfer efficiency in porcine cells.

### 2.2. Experiment 2

**Figure 2:**
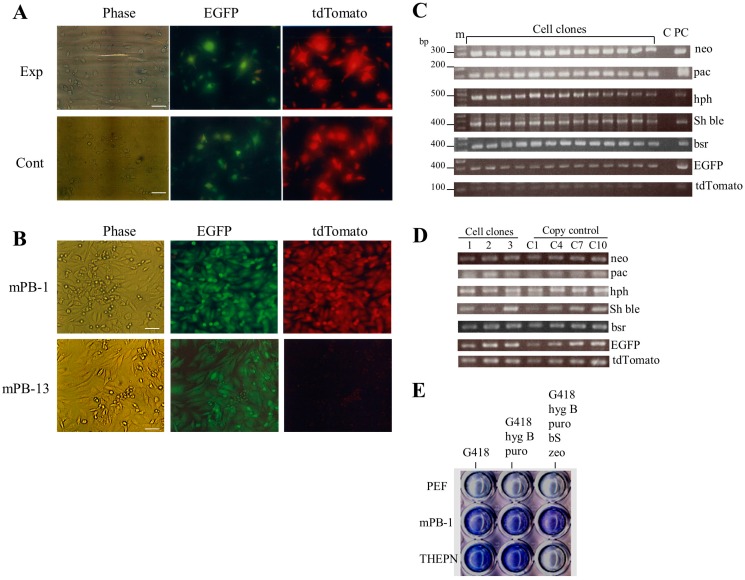
Acquisition of stable PEF transfectants after simultaneous transfection with seven *PB* vectors. (**A**) Fluorescence micrographs of PEFs one day after transfection in the presence (experimental group, Exp) or absence (control group, Cont) of pTrans. Note that in both transfection groups there are some cells exhibiting both green and red fluorescence, but no significant difference in gene transfer efficiency between these two groups was observed. Phase, taken under light; tdTomato, red fluorescence derived from *tdTomato* in pT-tdTomato; and EGFP, green fluorescence derived from *EGFP* in pT-EGFP. Scale bars = 20 µm; (**B**) fluorescence micrographs of stable PEF transfectants (m*PB*-1 and m*PB*-13). Note that m*PB*-1 exhibited both green and red fluorescence, whereas only green fluorescence was observed for m*PB*-13. The abbreviations above each figure are the same as shown in (**A**). Scale bars = 20 µm; (**C**) PCR analysis of stable PEF transfectants (numbered m*PB*-1 to 13). Genomic DNA isolated from each transfectant was subjected to regular PCR using the primer sets shown in the Materials and Methods. m, 100-bp ladder markers; lane C, normal PEFs; lane PC, control plasmids (pT-neo for detection of *neo*, pT-pac for detection of *pac*, pT-hph for detection of *hph*, pT-Sh ble for detection of *Sh ble*, pT-bsr for detection of *bsr*, pT-EGFP for detection of *EGFP* cDNA, and pT-tdTomato for detection of *tdTomato* cDNA); (**D**) determination of the number of copies of the introduced transposon DNA in the PEF transfectants (m*PB*-1 (lane 1), -2 (lane 2), and -3 (lane 3)). C1, C4, C7, and C10 indicate PEF DNA plus one, four, seven, or 10 copies of transposon DNA, respectively; (**E**) Assay of drug sensitivity in stable PEF transfectants. Cells (m*PB*-1, THEPN, and untransfected PEFs (PEF)) were plated in a 48-well plate, and cultured in medium containing G418, medium containing G418 + hygromycin B (hyg B) + puromycin (puro), or medium containing G418 + hyg B + puro + blasticidin S (bS) + zeocin (zeo), for 10 days. After culturing, cells were stained with Giemsa stain for visualization.

To demonstrate the utility of the *PB*-based gene delivery system for acquiring transfectants carrying multiple gene constructs, we transfected PEFs with a cocktail containing seven *PB* vectors (five drug resistant and two fluorescent plasmids) and pTrans (experimental group) or containing only seven *PB* vectors (control group). To assess transfection efficiency, fluorescence was inspected using a fluorescence microscope one day after transfection. No appreciable difference in the rate of cells with red and/or green fluorescence was noted (Exp vs. Cont in Figure 2A). However, the rate of stable colonies generated after drug selection was significantly different between the experimental and control groups. In the experimental group, there were 10–13 colonies, whereas no colonies were seen in the control group (Table 1). Inspection of fluorescence revealed that of 13 colonies tested, 12 had both red and green fluorescence (as exemplified by m*PB*-1 clone in Figure 2B), whereas the remaining colony expressed only green fluorescence (as exemplified by the m*PB*-13 clone in Figure 2B). These 13 colonies were subjected to colony isolation using paper methods as described in the Materials and Methods; all colonies were successfully propagated. PCR analysis of genomic DNA isolated from these colonies demonstrated that all colonies had five unique drug-resistance genes (Figure 2C). As expected, colonies (m*PB*-1 to m*PB*-12) with both red and green fluorescence had both *EGFP* and *tdTomato* cDNAs (Figure 2C). The m*PB*-13 colony exhibiting only green fluorescence had *EGFP*, but not *tdTomato* cDNA (Figure 2C).

Next, we examined the copy number of transposons integrated into the genome of stable PEF transfectants using clones m*PB*-1 to m*PB*-3; the results are shown in Figure 2D. Each clone was found to have 1–8 copies for each transposon per diploid cell (Table 2).

Furthermore, we tested whether the stable transfectants obtained exhibit their original phenotype after long-term cultivation in the absence of selective drug pressure. One clone (m*PB*-1) exhibiting both green and red fluorescence was cultivated in drug-free medium for three months and then cultured in medium containing all five selective drugs to examine the survivability of this clone. The THEPN cells (showing resistance against G418, puromycin, and hygromycin B; [23]), together with intact PEFs, were also cultured as controls. After 10 days of culture, cells were fixed and stained with Giemsa dye solution. The results are shown in Figure 2E. As expected, m*PB*-1 survived after treatment with five selective drugs, while the control THEPN and intact PEFs were killed by their treatment.

### 2.3. Mapping Insertion Sites by Splinkerette PCR

Splinkerette PCR was employed to map the insertion sites in the genome of the stable transfectant m*PB*-1 obtained in Experiment 2. Six recombinant clones each carrying different inserts were obtained. As shown in Table 3, there were at least five different *PB* insertion sites in those cells. Sequencing analysis demonstrated that *PB* was inserted exclusively into the TTAA target sites that had been duplicated upon insertion. All the insertion sites had adjacent sequences that were unrelated to the transposon vectors. BLAST searches of these adjacent sequences against the NCBI database demonstrated that they were all derived from known genomic sequences, including those from the porcine genome. These results suggest that *PB*-mediated integration into the genome occurred in the porcine cells.

### 2.4. Experiment 3: Testing of the Potential of the Transfected PEF Clone Using SCNT Technology

**Figure 3:**
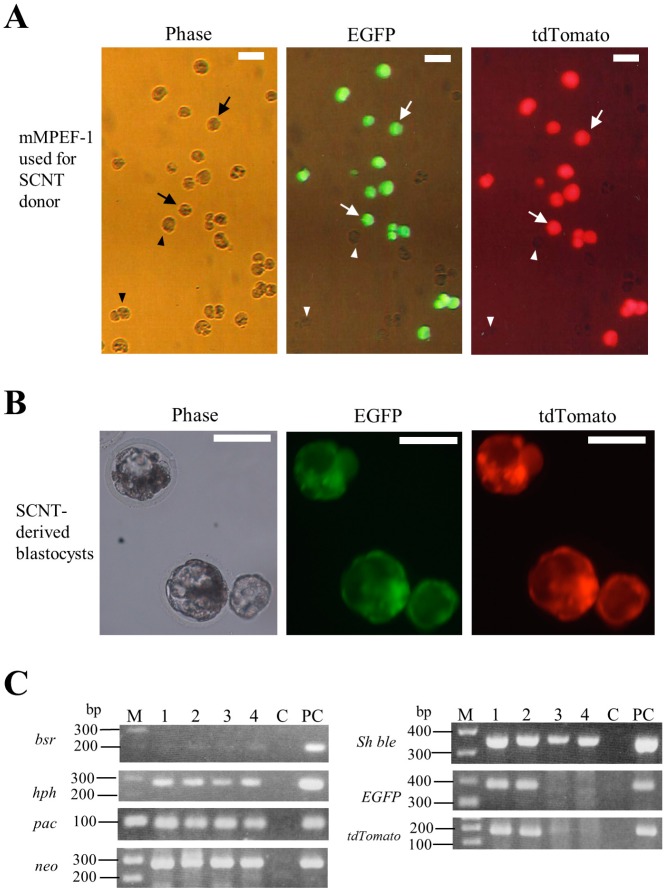
(**A**) Fluorescence micrographs of mMPEF-1 prior to SCNT. Note that there are some cells exhibiting both green and red fluorescence (arrowed), but other cells without any fluorescence (shown by arrowheads) in this clone. Phase, taken under light; tdTomato, red fluorescence derived from *tdTomato* in pT-tdTomato; and EGFP, green fluorescence derived from *EGFP* in pT-EGFP. Scale bars = 20 µm; (**B**) fluorescence micrographs of developing blastocysts derived from SCNT using mMPEF-1 as the SCNT donor. Phase, taken under light; tdTomato, red fluorescence derived from *tdTomato* in pT-tdTomato; and EGFP, green fluorescence derived from *EGFP* in pT-EGFP. Scale bars = 100 µm; and (**C**) PCR analysis of a single blastocyst derived from SCNT using mMPEF-1 as the SCNT donor. Genomic DNA isolated from each single blastocyst was subjected to WGA prior to PCR analysis. The data shown are the results from the nested PCR. M, 100-bp ladder marker; lanes 1 and 2, blastocysts showing both red and green fluorescence; lanes 3 and 4, blastocysts showing no fluorescence; lane C, normal PEFs; lane PC, control plasmids (pT-neo for detection of *neo*; pT-pac for detection of *pac*; pT-hph for detection of *hph*; pT-Sh ble for detection of *Sh ble*; pT-bsr for detection of *bsr*; pT-EGFP for detection of *EGFP* cDNA; pT-tdTomato for detection of *tdTomato* cDNA).

To demonstrate that the nuclei carrying multiple constructs prepared using the *PB*-based gene delivery system have the potential to develop as SCNT-treated embryos, microminipig-derived PEF clones termed as mMPEF were used as the nuclear donors. In a preliminary test, we found that nuclei from the transfected PEFs derived from a Clawn miniature pig as well as its intact parental cells were unable to promote development of SCNT embryos, probably due to their reduced developmental potential, which might have occurred during cell cultivation (data not shown). We, therefore, decided to use mMPEF as SCNT donors. We obtained a candidate clone (termed mMPEF-1) from microminipig-derived PEFs after transfection with seven *PB* vectors (five drug resistant and two fluorescent plasmids) and pTrans and subsequent selection with drugs. The microphotograph of mMPEF-1 is shown in Figure 3A. This clone had cells showing both red and green fluorescence and those with no fluorescence in a ratio of approximately 3:2. The latter cells may have all the drug resistance genes, but lack both fluorescent genes. SCNT using mMPEF-1 revealed that 18.1% (4/22) of the SCNT-treated embryos developed to normal blastocysts. As expected, 50% (2/4) of these embryos expressed both fluorescent markers (Figure 3B), but the other embryos did not fluoresce (data not shown). To confirm this event at the molecular biological level, they were singly isolated, lysed, and subjected to WGA. PCR analysis of these WGA samples demonstrated that blastocysts expressing both fluorescent markers had seven *PB* vectors (lanes one and two in Figure 3C), whereas those expressing no fluorescence lacked both fluorescent genes, but had five drug resistance genes (lanes three and four in Figure 3C). These results suggest that *PB*-mediated simultaneous integration of multiple constructs into the genome of porcine cells does not affect the development of cloned embryos.

## 3. Discussion

**Figure 4:**
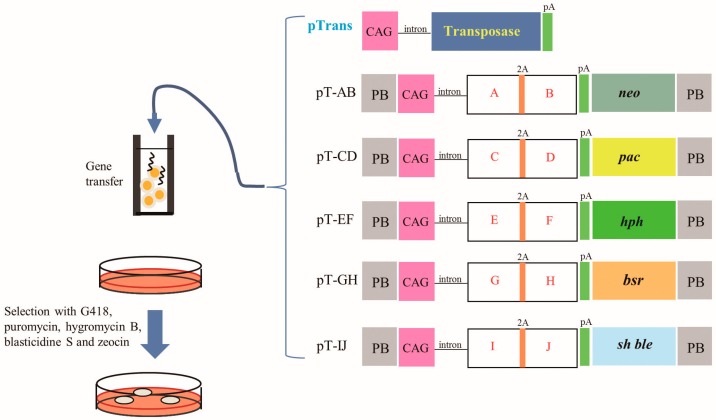
Future strategy to acquire porcine fibroblasts carrying multiple gene constructs using a *PB*-based gene delivery system. At least 5 plasmids (i.e., pT-AB, pT-CD, pT-EF, pT-GH and pT-IJ) can be used. In each plasmid, there is a unit conferring expression of specific drug resistance gene (i.e., *neo*) and a unit conferring expression of at least two types of proteins (i.e., A and B, C and D, E and F, G and H or I and J) under the control of upstream strong CAG-based promoter. Transfection of PEFs with these *PB* transposons and transposase expression vector pTrans and subsequent selection with multiple selective drugs will lead to generation of cells carrying multiple constructs in their genome.

There are several ways to introduce multiple gene constructs into the genome of domestic cells. One-step introduction of a single gene construct is one promising approach for obtaining cells with multiple genes of interest; in this approach, multiple genes are linked via internal ribosomal entry site (IRES) elements [24,25,26] or self-cleaving 2A peptide [27,28,29], and their expression is controlled by a single promoter. In fact, Carey et al. [30] succeeded in producing iPS cells using a single polycistronic vector carrying genes for 4 reprogramming factors. However, this method requires the assembly of expression units into a single vector, which is difficult to achieve and labor intensive. Sequential introduction of gene constructs is another way to obtain cells with multiple genes of interest, as shown in our previous work [23]. However, the repeated transfections required in this method cause cells to become refractory to transfection, leading to reduced transfection efficiency in later steps. Furthermore, this method requires a long time to acquire genetically modified cells with the desired constructs. One-step simultaneous introduction of multiple gene constructs appears to be a more realistic method, since it does not require a long time to select transfectants carrying multiple constructs. As shown in this study, simultaneous introduction of seven multigene gene constructs into a porcine cell can be achieved. Theoretically, it is possible to obtain a cell with at least 10 target genes in its genome, if five constructs carrying drug resistance gene expression units and an expression unit that confers two target genes using the 2A system are introduced into a cell at once along with the transposase expression plasmid, as depicted in Figure 4. However, the co-transfection of multiple single vectors has some drawbacks. It depends on the random integration of transgenes, which may lead to occasional transgene silencing and insertional mutagenesis. Notably, according to Wilson et al. [31], 67% of *PB*-derived transposon insertions occur in predicted transcriptional units, with 97% of these occurring within introns. Therefore, they can be considered useful as safe gene therapeutic reagents [32]. If our SCNT-derived embryos carrying seven transposons are allowed to develop to the full-term, the delivered cloned piglets should be a unique model to assess the safety problems associated with *PB*-mediated gene delivery.

In Experiment 3, in which PEFs carrying seven *PB* transposons were subjected to SCNT, we used PEFs derived from microminipig, because they have been shown to have the potential to promote the development of cloned embryos when intact microminipig-derived PEFs were used for SCNT as nuclear donors (under submission). Upon selection of PEF transfectants after *PB*-mediated gene delivery, both the isolated clones were heterogeneous concerning expression of fluorescence; some cells expressed both red and green fluorescence, but other cells did not, as shown in Figure 3A. In the cells showing no fluorescence, genes coding for fluorescent proteins might have been lost during transfection and subsequent selection with selective drugs. This was confirmed when SCNT-derived non-fluorescent blastocysts were examined at a molecular biological level. There were no fluorescent genes in those blastocysts, but drug resistant genes existed (see lanes three and four in Figure 3B). Efficient acquisition of cloned transgenic piglets requires enrichment of pure clones carrying target transgenes. Therefore, further purification of mMPEF-1 expressing both fluorescent markers through FACS-based cell selection or dilution-based cell cloning may be needed.

In this study, we clarified the usefulness of *PB* system for creating cloned transgenic piglets, because cloned embryos reconstituted with a PEF clone carrying seven transgenes in its genome successfully developed at least up to blastocysts in vitro. To date, only a few reports have demonstrated the use of the *PB* system in cells/early embryos from domestic animals. For example, Kim et al. [12] demonstrated that bovine fibroblasts transfected with *PB* vectors and a transposase expression vector contributed to generation of cloned embryos (blastocysts). Clark et al. [11] demonstrated that porcine cells can be efficiently transfected using the *PB*-based gene delivery system and can significantly improve the rate of transgenesis in vitro. Yi-bo et al. [33] and Kim et al. [34] transfected porcine fibroblasts with *PB* transposons carrying fluorescent genes and succeeded in generating cloned fluorescent embryos after SCNT of these transfected cells as SCNT donors. More interestingly, Wu et al. [35] reported successful production of cloned piglets using a strategy similar to ours. They introduced *PB* transposons into primary porcine fibroblasts and used these genetically modified cells as SCNT donors for the generation of transgenic cloned pigs. As a result, they obtained piglets showing red and green fluorescence constitutively. Li et al. [36] showed that it is possible to create transgenic pigs by cytoplasmic injection of *PB*-based pmhyGENIE-3 plasmid, a construct conferring a simple and self-inactivating plasmid into the in vivo (but not in vitro) fertilized pig zygotes. This *PB*-based gene delivery method appears to be a useful and effective way to create transgenic livestock. These reports, together with the present study, indicate that *PB* can be a useful, safe, and effective means to create genetically modified domestic animals.

## 4. Materials and Methods

### 4.1. Cell Lines and Culture

Clawn miniature pigs were purchased from Japan Farm, Ltd. (Kagoshima, Japan). PEFs were obtained from female fetuses on day 30 of pregnancy. Fetuses were also obtained from a microminipig [37] on day 30 of pregnancy. The procedure for cultivation of these cells is based Sato et al. [23]. For each experiment, cells passaged for 16–24 (Clawn miniature pig-derived PEFs) and 4–8 (microminipig-derived PEFs) generations were used.

The experiments described were performed in agreement with the guidelines of Kagoshima University Committee on Recombinant DNA Security and approved by the Animal Care and Experimentation Committee of Kagoshima University (no. 25036; dated on 8 August 2013).

### 4.2. Plasmids Carrying Selectable Marker Genes

*PB* expression vectors (Figure 1A) were generated using standard cloning procedures. Briefly, p*PB* (p*PB*-MCS-P5) is a vector carrying two *PB* acceptors with inverted repeats. pTrans (pCX-m*PB*) is a vector for expression of the *PB* transposase [38] under the chicken β-actin promoter-based promoter system (CAG; [39]). pT-neo (formerly referred to as p*PB*-PGKneo) is a p*PB*-based vector that carries a neomycin resistance gene (*neo*) expression unit (phosphoglycerate kinase (PGK) promoter + *neo* + poly(A) sites). pT-Sh ble (p029-1,2 (100921-6-1,5)) is a p*PB*-based vector that carries a zeocin resistance gene (*Sh ble*) expression unit (*SV40* early promoter + *Sh ble* + poly(A) sites). pT-hph (p030(101006-12-1)) is a p*PB*-based vector that carries a hygromycin B resistance gene (*hph*) expression unit (PGK promoter + *hph* + poly(A) sites). pT-bsr is a p*PB*-based vector that carries a blasticidin S resistance gene (*bsr*) expression unit (CAG promoter + *bsr* (derived from pCAG/bsr-7; [23]) + poly(A) sites). pT-pac is a p*PB*-based vector that carries a puromycin resistance (puromycin acetyltransferase) gene (*pac*) expression unit (CAG promoter + *pac* + poly(A) sites). pT-EGFP (pAZI) is a p*PB*-based vector that carries an enhanced green fluorescent protein (EGFP) expression unit (CAG promoter + *EGFP* cDNA + poly(A) sites). pT-tdTomato (pAZE) is a p*PB*-based vector that carries an expression unit of tandem dimer Tomato (*tdTomato*) cDNA under control of the CAG promoter.

### 4.3. Transfection for Obtaining Stable PEF Transfectants Carrying Multiple Transgenes

#### 4.3.1. Experiment 1

The efficiency of the *PB* system was tested with the PEFs derived from Clawn miniature pigs transfected using an electroporation-based Lonza Nucleofector system (Lonza Biologics, Cologne, Germany), because this system results in relatively high transfection efficiencies (>60%) in PEFs [40]. PEFs (4 × 10^4^) were electroporated in 100 µL of nucleofector solution (for primary fibroblasts) containing pT-pac + pTrans or pT-pac + pT-hph + pTrans (0.5 µg each) as an experimental group. The control group was transfected with the same plasmids without pTrans. After transfection, the cells were plated in a six-well plate (Iwaki Glass Co. Ltd., Tokyo, Japan) containing culture medium and incubated without selective drugs for five days. Six days after transfection, cells transfected with pT-pac + pTrans or pT-pac alone were cultured in the presence of puromycin. Similarly, cells transfected with pT-pac + pT-hph + pTrans or pT-pac + pT-hph were cultured in the presence of puromycin and hygromycin B. The drugs used in this study were purchased from Invitrogen Co. (Carlsbad, CA, USA), except for puromycin (InvivoGen Inc., San Diego, CA, USA). Concentrations of hygromycin B and puromycin were 40 and 2 µg/mL, respectively, based on data from Sato et al. [23]. Ten days after selection, cells were fixed with 4% paraformaldehyde (PFA) in Dulbecco’s modified phosphate-buffered saline without Ca^2+^ and Mg^2+^ (D-PBS) for 10 min at room temperature. Giemsa staining was performed to count the number of emerging colonies (containing 300–700 cells) using a Giemsa staining kit (Wako Pure Chemical Industries, Ltd., Osaka, Japan). After staining and subsequent washing with water, the cells in each well were photographed under light.

#### 4.3.2. Experiment 2

For acquiring stable transfectants carrying the seven multigene constructs listed in Figure 1A (except for pTrans), PEFs (2 × 10^5^) were transfected with a total of eight circular plasmids (0.5 µg each; Figure 1A) using the transfection system described in Experiment 1; these transfectants served as the experimental group. For the control group, the same seven multigene constructs used in the experimental group without pTrans were transfected. After transfection, the cells were plated in a 100-mm gelatin-coated dish (Iwaki Glass Co. Ltd.) and incubated in culture medium without selective drugs for 5 days. During this time, fluorescence was inspected and recorded using a fluorescence microscope, as described below. Six days after transfection, cells were cultured with five selective drugs from Invitrogen (G418 (400 µg/mL), puromycin, hygromycin B, blasticidin S (8 µg/mL), and zeocin (800 µg/mL) based on results from Sato et al. [23]). Ten days after selection, the emerging colonies were assessed for expression of fluorescence under a fluorescence microscope as described below and then picked using a small Whatman paper disc (approximately 3 mm in diameter), which had been dipped in 0.125% trypsin/0.01% EDTA, as described previously [23]. The discs carrying cells were transferred directly to wells of a gelatin-coated 48-well plate (Iwaki Glass Co. Ltd.) with selective drug-containing culture medium. These cells were cultured for 20–30 days until they reached confluency, and were further propagated in a stepwise manner and checked for fluorescence and the presence of the introduced exogenous constructs, as described below. Isolation of genomic DNA was based on the method of Sato et al. [41], dissolved in 50 μL of sterile water, and stored at 4 °C.

#### 4.3.3. Experiment 3

The PEFs derived from microminipigs were transfected with eight circular plasmids (including *PB* transposase expression plasmid pTrans; 0.5 µg each; Figure 1A), according to the method described in experiments 1 and 2. Selection with five selective drugs and subsequent colony isolation was the same as described in Experiment 2.

SCNT was performed according to the method of Sato et al. [42] and Miyoshi et al. [43,44]. Each nucleus from the microminipig-derived PEF clones (hereafter referred to as mMPEF) expressing both red and green fluorescence was introduced into a single enucleated oocyte using micromanipulators. The development of SCNT treated embryos was evaluated by the rates of cleavage and blastocyst formation at two and seven days of culture, respectively. The developing blastocysts were assessed for expression of red or green fluorescence under a fluorescence microscope, as described below. Then, they were fixed in 4% PFA in D-PBS for 30 min at room temperature and stored at 4 °C in D-PBS covered with paraffin oil in a Terasaki microtest plate (Nunc, Roskilde, Denmark) prior to molecular biological analysis.

For isolation of genomic DNA, the single fixed blastocyst was transferred to a PBS drop (1 μL) in a 0.5-mL PCR tube (#PCR-05-C.; AxyGen Scientific, Inc., Union City, CA, USA) with the aid of a mouthpiece-controlled micropipette. Genomic DNA was extracted according to the method described by Sato et al. [41], dissolved in 20 μL of sterile water, and stored at 4 °C. In order to increase the total genomic DNA amount, we employed whole genome amplification (WGA) using the Illustra GenomiPhi V2 DNA Amplification kit (#25-6600-31; GE Health Care Japan, Tokyo, Japan) as previously described [41,45]. The resulting WGA products (2 μL) were subjected to PCR and then to nested PCR, as mentioned below.

### 4.4. Detection of Fluorescence

Fluorescence in the cells was examined with an Olympus BX60 fluorescence microscope (Olympus, Tokyo, Japan) with DM505 (BP460-490 and BA510IF; Olympus) and DM600 filters (BP545-580 and BA6101F; Olympus), which were used for *EGFP*-derived green fluorescence and *tdTomato*-derived red fluorescence, respectively. Microphotographs were taken using a digital camera (FUJIX HC-300/OL; Fuji Film, Tokyo, Japan) attached to the fluorescence microscope and printed using a Mitsubishi digital color printer (CP700DSA; Mitsubishi, Tokyo, Japan).

### 4.5. PCR Analysis

PCR amplification reactions using genomic DNA (~5 ng) isolated from PEFs were performed in a total volume of 20 µL, based on the protocol described by Sato et al. [23]. The primer sets used are based on our previous paper [23]. With each primer set, the presence of pT-neo, pT-pac, pT-hph, pT-bsr, pT-Sh ble, and pT-EGFP can be detected as 297, 110, 443, 289, 375, and 400 bp bands, respectively. For detection of pT-tdTomato, primer set TDR-3S (5′-CCC GTA ATG CAG AAG AAG ACC-3′) and TDR-3RV (5′-GTG ATG TCC AGC TTG GTG TCC-3′) were used, which yields a 206-bp product from the middle region of tdTomato cDNA (accession no. AY678269). As a negative control, 0.5 µg of genomic DNA from the untransfected PEFs was used. As a positive control, 5 ng of each plasmid listed in Figure 1A were used. For detection of the introduced genes in the SCNT-treated blastocysts, nested PCR was performed using the same conditions from the 1st PCR using l µL of the first PCR product and a primer set (Ne-5S (5′-CTG TTC GCC AGG CTC AAG GCG-3′)/Ne-5RV (5′-CTC GTC AAG AAG GCG ATA GAA-3′) for detection of *neo*, puro-2S (5′-TCT ACG AGC GGC TCG GCT TCA-3′)/puro-4RV (5′-TCA GGC ACC GGG CTT GCG GGT-3′) for detection of *pac*, Hyg-4S (5′-GTC TTG CAA CGT GAC ACC CTG-3′)/Hyg-2RV (5′-GAA GTT TCT GAT CGA AAA GTT-3′) for detection of *hph*, bsr-2S (5′-AAG ATT ACA ATG CTT TAT GAG-3′)/bsr-2RV (5′-TGT CTA ACA GCT ACA ATC GTG-3′) for detection of *bsr*, ble-2S (5′-TTG ACC AGT GCC GTT CCG GTG-3′)/ble-2RV (5′-CTC CTC GGC CAC GAA GTG CAC-3′) for detection of *Sh ble*, EGFP-10S (5′-CCT GAA GTT CAT CTG CAC CAC-3′)/EGFP-10RV (5′-GTT GTG GCG GAT CTT GAA GTT-3′) for detection of *EGFP* cDNA, or TDR-4S (5′-AAG AAG ACC ATG GGC TGG GAG-3′)/TDR-4RV (5′-AGC TTG GTG TCC ACG TAG TAG-3′) for detection of *tdTomato* cDNA). With each primer set, the presence of pT-neo, pT-pac, pT-hph, pT-bsr, pT-Sh ble, pT-EGFP, and pT-tdTomato can be detected as 279, 92, 271, 194, 357, 384, and 185 bp bands, respectively.

The PCR products were separated by electrophoresis on 1.5% (*w*/*v*) agarose gels, stained with ethidium bromide (EtBr), and visualized using a UV transilluminator.

For estimating the number of copies of the introduced transposons in the PEF transfectants by PCR analysis, ~5 ng of genomic DNA was subjected to PCR using the same primer sets used for the characterization of the PEF transfectants (m*PB*-1 to m*PB*-13). The conditions for the PCR were the same as those used previously, except that the annealing temperature was reduced to 46 °C to 44 °C. Two microliters of the PCR product was then electrophoresed on agarose gels along with the PCR products derived from serially diluted transposon DNA, stained with EtBr, and photographed. In this case, 7–7.5 fg of the transposon DNA was calculated to be equivalent to one copy of the DNA per diploid cell. Based on this calculation, serially diluted transposon DNA was added to the PEF (non-treated) DNA (one, four, seven, and 10 copies per diploid cell) and processed concomitantly as for the experimental samples. Quantitation of the PCR bands in the agarose gels was performed using ImageJ software (Available online: http://rsbweb.nih.gov/ij/download.html). The concentration of each band was determined by using a standard curve to compare the experimental samples.

### 4.6. Confirmation of Multi-Drug Resistance in Transfectants Carrying Multiple Constructs

Cells containing *tdTomato* (T), *hph* (H), *EGFP* (E), *pac* (P), and *neo* (N) were termed THEPN cells. Stable PEF transfectants (derived from Clawn miniature pig) carrying all the transposon plasmids listed in Figure 1A, THEPN cells exhibiting resistance against G418, hygromycin B, and puromycin [23], and untransfected PEFs were recovered from dishes by trypsinization. These cells (~3 × 10^4^) were plated directly in wells of a gelatin-coated 48-well plate one day before the addition of drugs. Selection was performed for seven days by incubating cells in medium containing G418, medium containing G418 + hygromycin B + puromycin, or medium containing G418 + hygromycin B + puromycin + blasticidin S + zeocin. Then cells were subjected to Giemsa staining to evaluate cell growth. After staining and subsequent washing with water, the cells in each well were photographed under light.

### 4.7. Mapping Insertion Sites by Splinkerette PCR

Splinkerette PCR was performed to map *PB* integration sites in the transfectants, according to Potter and Luo [46] and based on the manufacturer’s protocol (Splinkerette Protocol; Available online: http://www.cmhd.ca/protocols/genetrap_pdf/Splinkerette%20Protocol%20Single%20Clone.pdf#search=‘splinkerette’). *Sau3*AI digested genomic DNA was ligated using a splinkerette adapter generated by annealing HMSpAa and HMSpBb. Junction fragments were PCR amplified using primers HMSp1 and *PB*-R-Sp1. Nested PCR was performed using primers HMSp2 and *PB*-L-Sp2. PCR products were cloned into the TA-cloning vector pCR2.1 (Invitrogen Co.) and sequenced with standard primers.

### 4.8. Statistical Analysis

The data obtained from Experiment 1 were statistically analyzed using GraphPad PRISM 5 for Windows software (GraphPad Software, Inc., La Jolla, CA, USA). Data were analyzed by one-way repeated ANOVA and expressed as mean ± SD for three independent experiments. Statistical significance was determined by Student’s *t*-test. *p-*Values < 0.05 were considered statistically significant.

## 5. Conclusions

We achieved simultaneous transfection of PEFs with at least seven multigene gene constructs using the *PB*-based gene delivery system. The resulting PEF clones carrying the transgenes had the ability to proceed through development of cloned embryos when they were used as SCNT donors. Notably, Wilson’s group [31,32] demonstrated that most transposons after *PB*-mediated gene delivery are integrated into the intronic region of the host genome, suggesting the suitability of this gene delivery system for human gene therapy. In this context, the *PB* will be useful as a powerful and safe genetic tool for producing cloned transgenic pigs that potentially confer pig-to-human xenotransplantation, as well as the creation of genetically modified disease-model pigs.

## Figures and Tables

**Table 1:** Summary of Experiment 2.

Treatment	No. of Stable Transfectants	Fluorescence Expression ^1^		
		R/G	R	G
Experimental group				
1	13	12	0	1
2	10	10	0	0
3	18	16	0	2
Control group				
1	0	–	–	–
2	0	–	–	–
3	0	–	–	–

^1^ PEFs (2 × 10^5^) were transfected in a nucleofector solution containing seven *PB* vectors and pTrans (Experimental group), or only seven *PB* vectors without pTrans (Control group). After selection with multiple selection drugs, the emerging colonies were picked and propagated as stable transfectants. They were then examined for fluorescence expression under a fluorescence microscope and classified as R/G (cells expressing both EGFP and tdTomato fluorescence), R (cells expressing tdTomato alone), and G (cells expressing EGFP alone). Transfection was performed three times.

**Table 2:** Estimation of the copy number of transgenes in the PEF transfectants ^1^.

Transfectants	pT-EGFP	pT-tdTomato	pT-neo	pT-pac	pT-bsr	pT-hph	pT-Sh ble
m*PB*-1	4	2	2	2	1	4	3
m*PB*-2	7	7	5	8	4	2	1
m*PB*-3	6	5	7	4	3	2	7

^1^ Estimation of the copy number of each transgene integrated into the host genome of PEF transfectants was performed by PCR using serially diluted transposon DNA, as described in the Materials and Methods section. Identification of each transposon was performed using the following primer sets [23]: EGFP-7S/EGFP-7RV for pT-EGFP, TDR-3S/TDR-3RV for pT-tdTomato, Ne-4S/Ne-4RV for pT-neo, puro-S/puro-3RV for pT-pac, bsr-S/bsr-RV for pT-bsr, Hyg-3S/Hyg-RV for pT-hph, and ble-F/ble-B for pT-Sh ble.

**Table 3:** Splinkerette PCR analysis results indicating *PB*-mediated integration sites in the porcine genome.

No.	Sequence Corresponding to Endogenous Porcine Genome (5′−3′) ^1^	Known Sequences Showing Similarity ^2^ to Endogenous Porcine Genome
1	**TTAA**AATAAGCATTGAAAAGACTTAGAAGTTGGGAAC	*Rattus norvegicus* clone CH230-115B16, (99, 310/312)
	GCTCAGCACGCGTCAATCTAAAAGTGGTTTTGGTTTC	
	ATCTGGACAAGCCCATGAG	
2	**TTAA**AAAGATGCAATATGGATTTTAACAGAGGTGTCT	*Rattus norvegicus* clone CH230-102O7, (99, 168/169)
	TAAGACAATAGGCCCTTTTAGCATCTATTGTGAGGCT	
	GGCTCTGCCTTGCTGGTTT	
3	**TTAA**GCACATTAGGCACATTTAGAGACGTTTGTCTGT	*Rattus norvegicus* clone CH230-75C3, (98, 83/85)
	AGCATCCTCCATAATTTATAATGGATTTACAACCAAA	
	CTGTAAACAATA	
4	**TTAA**GAACCTTTAGCTAGCATGGCGGCCGAAAAGAAC	*Rattus norvegicus* clone CH230-334F16, (100, 80/80)
	CCGCTCCCCGCCTCCCAGGAGCTTCTGATTGGACAAC	
	CTGCCT	
5	**TTAA**ACAGATTGTTTATCTTCCTCCAGCGAGCACAAA	*Rattus norvegicus* clone CH230-81E19, (100, 171/171)
	ACGCCATGCCGAAATGGGAACCAGATTTTTCTACTCA	
	GTGAACTCCGTGTGGTTTC	

^1^ The TTAA sequence recognized by the transposase during both excision and integration is shown by bold; ^2^ Sequence similarity is shown below as a percentage (number of nucleotides from the query per number of nucleotides from known gene) in parentheses.
